# The effect of matrine and glycyrrhizic acid on porcine reproductive and respiratory syndrome virus in Vitro and in vivo

**DOI:** 10.1186/s12985-024-02415-w

**Published:** 2024-07-04

**Authors:** Zhilong Zhang, Wenyi Wu, Qiannan Li, Fangfang Du, Xuebing Wang, Mingfan Yang, Hongying Zhang

**Affiliations:** 1 College of Veterinary Medicine, Henan Agricultural University, Zhengzhou city, Henan P.R. China; 2 Key Laboratory for Animal-Derived Food Safety of Henan Province, Zhengzhou city, Henan P.R. China; 3 Ministry of Education Key Laboratory for Animal Pathogens and Biosafety, Zhengzhou city, Henan P.R. China

**Keywords:** Matrine, Glycyrrhizic acid, Porcine reproductive and respiratory syndrome virus, Antiviral activity against PRRSV

## Abstract

Porcine reproductive and respiratory syndrome (PRRS) is endemic worldwide, seriously affecting the development of the pig industry, but vaccines have limited protective effects against PRRSV transmission. The aim of this study was to identify potential anti-PRRSV drugs. We examined the cytotoxicity of seven compounds formulated based on the mass ratio of glycyrrhizic acid to matrine and calculated their inhibition rates against PRRSV in vitro. The results showed that the seven compounds all had direct killing and therapeutic effects on PRRSV, and the compounds inhibited PRRSV replication in a time- and dose-dependent manner. The compound with the strongest anti-PRRSV effect was selected for subsequent in vivo experiments. Pigs were divided into a control group and a medication group for the in vivo evaluation. The results showed that pigs treated with the 4:1 compound had 100% morbidity after PRRSV challenge, and the mortality rate reached 75% on the 8th day of the virus challenge. These results suggest that this compound has no practical anti-PRRSV effect in vivo and can actually accelerate the death of infected pigs. Next, we further analyzed the pigs that exhibited semiprotective effects following vaccination with the compound to determine whether the compound can synergize with the vaccine in vivo. The results indicated that pigs treated with the compound had higher mortality rates and more severe clinical reactions after PRRSV infection (*p* < 0.05). The levels of proinflammatory cytokines (IL-6, IL-8, IL-1β, IFN-γ, and TNF-α) were significantly greater in the compound-treated pigs than in the positive control-treated pigs (*p* < 0.05), and there was no synergistic enhancement with the live attenuated PRRSV vaccine (*p* < 0.05). The compound enhanced the inflammatory response, prompted the body to produce excessive levels of inflammatory cytokines and caused body damage, preventing a therapeutic effect. In conclusion, the present study revealed that the in vitro effectiveness of these agents does not indicate that they are effective in vivo or useful for developing anti-PRRSV drugs. Our findings also showed that, to identify effective anti-PRRSV drugs, comprehensive drug screening is needed, for compounds with solid anti-inflammatory effects both in vitro and in vivo. Our study may aid in the development of new anti-PRRSV drugs.

## Introduction

Porcine reproductive and respiratory syndrome (PRRS) is caused by porcine reproductive and respiratory syndrome virus (PRRSV) and is one of the most common infectious diseases affecting the pig industry worldwide [1]. The clinical manifestations of PRRS are reproductive dysfunction in sows, respiratory diseases in growing pigs, and slow growth and high mortality rates in weaned pigs, collectively causing enormous economic losses in pig production [2, 3]. PRRSV is a small enveloped RNA virus with a linear, single-stranded, sense genome and belongs to the *Arteriviridae* family of the order *Nidovirales* [4]. PRRSV is internalized into host cells through interactions between PRRSV proteins and cellular receptors. Upon viral invasion of the cell, the host’s antiviral immune system is rapidly activated to inhibit viral replication. To remain host-adapted, some viruses have evolved a variety of sophisticated strategies to manipulate the host machinery and circumvent the host’s antiviral response [5]. Currently, vaccination strategies, including those in which live-attenuated vaccines, modified live viruses vaccines, DNA vaccines and immune-adjuvanted vaccines are used are the most cost-effective means to prevent PRRS [6–8]. However, due to the diversity of epidemic strains, these vaccines are incapable of producing sustainable disease prevention and control, and safer and more efficient vaccines have not been developed that have advanced to the clinical stage [9, 10]. Research on new vaccines and antiviral drugs is needed to identify effective methods for the prevention and control of PRRS, which continues to be a high priority in the global pig industry [11].

Traditional Chinese medicines (TCMs) contain very complex chemical components and have various clinical applications. Many natural products and their derivatives have been confirmed to have natural antiviral effects [12]. TCMs are rich in resources, have few drug residues, and can enhance the immunity. With the development of advanced molecular and analytical techniques, more antiviral and immunomodulatory effects of TCMs and their derivatives, such as glycosides, terpenoids, isoflavones, alkaloids, and asiatic acid, have been discovered and clarified [11, 13–15]. Currently, studies have confirmed that several bioactive compounds extracted from TCMs have potent antiviral activities against PRRSV in vitro based on different antiviral strategies [16, 17], including those that prevent the viruses from attaching to putative receptors or blocking their entrance into cells in vitro by interfering with the process that leads to the uncoating of the viral genome. (–)-Epigallocatechin-3-gallate (EGCG), a natural bioactive compound isolated from green tea [18], has multiple functions, including inhibiting PRRSV replication in MARC-145 cells in vitro [11]. Curcumin is the most abundant bioactive compound in the rhizomes of *Curcuma longa*, and it has been reported to inhibit the entry of PRRSV into MARC-145 and PAM cells by interfering with all postinternalization stages of PRRSV [19].

The components of TCMs that exert antiviral effects include both individual components and groups of components [20–22]. OCD20015-V009 is an herbal mix of water-extracted Ginseng Radix, Poria (Hoelen), Rehmanniae Radix, Adenophorae Radix, Platycodi Radix, Crataegii Fructus, and Astragali Radix [23], and chlorogenic acid and ginsenoside Rd are the dominant components that stimulate the antiviral response in murine macrophages and mice exposed to viral infections. Many traditional Chinese medicine monomers or compound preparations have multichannel antiviral effects in vitro, and good antiviral effects can be achieved at safe concentrations within a prescribed dosage range. Matrine has antitumor, antiarrhythmic, anti-inflammatory, and antiviral effects [24]. Matrine has an excellent antiviral effect when used alone. When combined with other antiviral drugs, it can exert multiple antiviral actions, with the overall antiviral effect being more noticeable [25]. Glycyrrhizic acid (GL) is a broad-spectrum antiviral ingredient with inhibitory effects on many viruses [14]. Previous studies on the anti-PRRSV effects of glycyrrhizic acid and matrine monomers in our laboratory showed that matrine and glycyrrhizic acid monomers both had significant direct killing and therapeutic effects on RRSV. Because most research on the anti-PRRSV effects of Chinese medicines has involved in vitro experiments, it remains to be determined whether these medicines can also exert their antiviral effects in vivo. Based on previous work, the present study used matrine and glycyrrhizic acid monomers to form compounds of different mass ratios and tested them in vitro. Compounds superior to other TCM monomers in terms of antiviral effects were screened and used in in vivo tests. The antiviral ability of the optimal compound ratio was evaluated in vivo based on clinical symptoms and serum antibody levels, and the synergistic enhancement of the optimal compound ratio on the vaccine was evaluated based on clinical symptoms and serum antibody and cytokine levels to identify candidate drugs for the treatment of PRRSV.

## Materials and methods

### Reagents and antibodies

Glycyrrhizic acid (Lot NO: 16,032,902) and matrine (Lot NO: 15,082,608) were obtained from Jingkehuaxue Co., Ltd. (Shanghai, China), and ribavirin (Lot NO: 160,930) was obtained from Baili Pharm Co., Ltd. (Sichuan, China). For in vitro experiments, glycyrrhizic acid, matrine and ribavirin were stored in cell maintenance fluid, and the samples were stored in pure water for in vitro experiments. Dulbecco’s modified Eagle’s medium (DMEM) and fetal bovine serum (FBS) were purchased from Invitrogen Gibco Inc. (Carlsbad, CA, United States). Penicillin (100 U/ml), streptomycin (0.1 mg/ml), BSA, MTT and DMSO were purchased from Solarbio (Beijing, China). DAPI was purchased from LeiGen (Beijing, China), and anti-PRRSV N (MAb SDOW17-FITC, Lot NO: 1211011F7) was purchased from the Rural Technology Initiative (Beijing, China).

### Cell culture, virus strain and vaccine

MARC-145 cells and the PRRSV JXA1 strain were obtained from the Key Laboratory of Animal Immunology, Henan Academy of Agricultural Sciences, and the cells were cultured in high-glucose DMEM supplemented with 10% FBS with antibiotics (100 U/ml penicillin and 0.1 mg/ml streptomycin) and maintained in a 37 °C, 5% CO_2_ incubator. The highly pathogenic porcine reproductive and respiratory syndrome live vaccine (JXA1-R) was provided by Puike Bioengineering Co., Ltd. (Lot NO: 11CW1804006).

### Experimental animals

A total of 28 commercial pigs aged 48–50 days and weighing 5–7 kg were obtained from the National Veterinary Drug Engineering Research Center of Luoyang City, Henan Province. Routine tests were negative for porcine circular antigen and PRRSV antigen and antibody. Pig feed was purchased from Henan Tiankang Hongzhan Industrial Co., Ltd. (product number: Q/HTK 011-2018). All animal care and experimental protocols were reviewed and approved by the Henan Provincial Key Laboratory of Animal Immunology, Henan Academy of Agricultural Sciences (Zhengzhou, China, approval no. SYXK (Henan2014-0007)).

### Determination of safe drug concentration

The density of digested MARC-145 cells was adjusted to 4 × 10^5^ cells/mL, and the cells were mixed evenly, added to 96-well cell plates (100 µL per well), and placed in a 37 °C, 5% CO_2_ incubator for culture. After the formation of a monolayer, the seven compound drugs and ribavirin were diluted with cell maintenance solution to an initial concentration of 2 mg/mL, then sequentially diluted to create 3 concentrations (2-fold) with maintenance solution and added to the long-term growth medium. On the MARC-145 cell monolayer, 100 µL of each compound was added per well. Each dilution was replicated in 6 wells. The blank and cell control were set up and placed in a 37 °C, 5% CO_2_ incubator for 72 h. After 4 h, 10 µL of MTT solution was added to each well, and the culture was continued. After 4 h, the culture plates were removed, and the supernatant was discarded. DMSO (100 µL) was added to each well, the wells were shaken on a microplate reader for 10 min, and the OD value at 492 nm was recorded. The inhibition rate of drugs on cells was calculated based on the cytopathic equation. The maximum safe concentration (TC_0_) was the maximum drug concentration at which the cytopathic rate was less than 10%. The median inhibitory concentration (TC_50_) of the drugs was calculated by the probit regression method using SPSS software.

Cytopathic rate (%)=(OD cell control – OD drug control)/OD cell control group ×100.

### Determination of the median lethal dose (TCID _50_) of PRRSV

One hundred microliters of virus stock solution was diluted 10-fold with maintenance solution to create 8 concentrations. MARC-145 cells with adjusted cell numbers were plated in 96-well plates. After the cells grew to a monolayer, 100 µL of virus solution was added to each well. Six replicate wells were used for each concentration, and a cell control and blank control were used. The 96-well plate was incubated in a 37 °C, 5% CO_2_ incubator for 1.5 h, the viral solution was discarded, the wells were washed twice with PBS, 100 µL of maintenance solution was added for an additional 72 h, and 10 µL was added 4 h before the end of the culture. After MTT was added for another 4 h, the 96-well plate was removed, the supernatant was discarded, and 100 µL of DMSO was added to each well. The wells were shaken on a microplate reader for 10 min, and the OD at 492 nm was recorded. The cytopathic rate of each well was calculated according to the formula:

Cytopathic rate=(OD cell control-OD virus control)/(OD cell control-OD blank control)×100.

The number of diseased wells in each group was recorded, a rate greater than 10% was considered cytopathic. The Reed-Muench method was used to calculate the PRRSV TCID_50_ as follows: distance ratio = (percentage above 50% − 50%)/(percentage above 50% - percent below 50%), and the logarithm of TCID _50_ = virus dilution above 50% logarithm of + distance proportion × logarithm of the dilution factor.

### Time-of-addition assay

Matrine and glycyrrhizic acid monomers were diluted to two concentrations, 0.5 mg/mL and 0.25 mg/mL, which were used as monomer controls. Matrine and glycyrrhizic acid were mixed at ratios of 1:1, 1:2, 1:3, and 1:4 and diluted 2-fold with maintenance solution to create three concentrations from 0.5 mg/mL. For mixing ratios of 2:1, 3:1, and 4:1, the maintenance solution was diluted to create 3 concentrations from 1 mg/mL to form the drug group.

The number of digested MARC-145 cells was adjusted to 4 × 10^5^ cells/mL, and the cells were mixed evenly, added to 96-well plates at 100 µL per well, and cultured in a 37 °C, 5% CO_2_ incubator. After the cells grew into a monolayer, the growth medium was discarded, and the cells were washed twice with PBS. A mixture of matrine and glycyrrhizic acid in different ratios was added before (pretreatment), during (combination therapy), or after PRRSV infection (posttreatment). In addition, a cell control, a virus control, a glycyrrhizate monomer control and a matrine monomer control were established. For pretreatment, the cells were incubated at 37 °C for 4 h, washed 3 times with PBS, and then infected with PRRSV for 2 h. For cotreatment, the cells were simultaneously incubated with PRRSV and drugs at 37 °C. After 2 h, the virus and drugs were removed, and the cells were washed three times with PBS. Posttreatment, the cells were first infected with PRRSV for 2 h at 37 °C and then coincubated in fresh medium containing the drug for 68 h. Ten microliters of MTT was added to each well 4 h before the end of culture. After further culturing for 4 h, the cell plate was removed, the supernatant was discarded, and 100 µL of DMSO was added to the plate in the dark. The wells were shaken on a microplate reader for 10 min, and the optical density (OD) was measured at 492 nm. Using the viral inhibition rate, the half-maximal inhibitory concentration (ED_50_) of the virus was calculated by the SPSS software probit regression method, and the therapeutic index was obtained: (TI) TI = TC_50_/ED_50_.The drug inhibition rate was calculated as follows:

Drug inhibition rate (%)=(OD administration group-OD virus control group)/(OD cell control group-OD virus control group)×100.

### Effect of the optimal compound on virus replication

After MARC-145 cells grew into a monolayer in 96-well plates, the growth medium was discarded, the wells were washed twice with PBS, and 100 µL of virus solution at 100 TCID_50_ was added to each well. After incubation for 2, 12, 24, or 36 h, the virus solution was discarded, and the cells were washed with PBS for 2 times. The optimal compound drug was diluted 2-fold with maintenance solution to make 4 concentrations, and 100 µL of the drug was added to each well. Simultaneously, a cell control and virus control were set up and cultured for 68 h. Next, 10 µL of MTT was added to each well and incubated for another 4 h, after which the 96-well plates were removed. After the supernatant was discarded, 100 µL of DMSO was added to each well, the wells were shaken on a microplate reader for 10 min, and the OD at 492 nm was recorded.

### Indirect IFA

The adjusted number of cells was plated on 6-well plates, which were covered with cell membranes. The cells were included in the control, virus control, 0.25 mg/mL compound, and 0.125 mg/mL ribavirin groups. After incubation at 37 °C for 72 h, the cells were removed, washed three times with PBST for 5 min each, fixed in methanol-acetone solution at a volume ratio of 1:1 at -20 °C for 20 min, washed three times again with PBST for 5 min each, and incubated with 1% ethoxylate (PBST) for 20 min at -20 °C. The cells were blocked with BSA for 1 h at room temperature, removed and placed on glass slides. Next, 300 µL of PRRSV N antibody diluted 1:30 was added, and the mixture was placed in a wet box for incubation at 37 °C in the dark for 2 h and then washed three times with PBST. Cell nuclei were stained by adding 200 µL of DAPI. After being protected from light for 5 min, the cells were washed three times with PBST, and the sections were mounted with mounting medium and observed under a fluorescence microscope in the dark.

### Clinical symptom detection

Eight pigs were randomly divided into 2 groups: the control group and the medication group. Each pig was injected intramuscularly with 3 mL of the PRRSV JXA1 strain virus solution with a virulence titer of 10^5.5^ TCID_50_/mL. The medication group started medication on the 2nd day after challenge, receiving an intramuscular injection once a day for 7 days at a dose of 25 mg/kg. The control group was injected with the same dose of normal saline.

The control group and the treatment group were housed in separate rooms to prevent cross-infection. The pigs were observed daily after challenge to record whether the food intake of the pigs was normal and whether the pigs had symptoms such as poor breathing, cough, eyelid swelling, loss of appetite, runny nose, diarrhea, or depression. The appearance of clinical symptoms indicated disease, and the morbidity and mortality of the pigs were calculated based on the clinical symptoms of the pigs. The calculation formulae are as follows:

Incidence rate = number of diseased animals/number of animals × 100.

Mortality = number of dead heads/number of animals × 100.

During the experiment, starting from 2 days before challenge, the body temperature of the pigs was measured at 8:00 am and 1:00 pm each day, and the temperatures were averaged. A temperature greater than 40 °C was considered indicative of fever.

During the entire experiment, the feed consumed by each group was recorded daily, the pigs in each group were weighed on Days 0, 7, 14, and 21, and the weight gain of the pigs was calculated.

### Detection of serum antibody levels

Blood collected from the porcine anterior vena cava on Days 0, 7, 14, 21, 24, 26, 28, 30, 35 and 42 of the experiment was placed in a 37 °C incubator for 1 h, followed by centrifugation at 5000 r for 4 min at -20 °C to separate the serum. The S/P value of the PRRSV N protein antibody in pigs was detected using the IDEXX PRRS X3 antibody detection kit from IDXX Co., Ltd. (USA, J661). Effectiveness was defined as follows: positive mean value – negative mean value ≥ 0.150.

The S/P was calculated as follows: S/P=(OD value of sample 650-negative control)/(mean value of positive control-mean value of negative control).

A value of S/*P* < 0.4 was considered negative, and a value of S/*P* ≥ 0.4 was considered positive.

### Determination of the synergistic immune effects of the compound drug vaccine

Twenty pigs were randomly divided into 4 groups of 5 pigs: the immunization group (IM), immunization control group (IMC), nonimmunization group (NIM), and nonimmunization control group (NIMC). The IM and IMC groups were immunized with a live HPRS vaccine (JXA1-R strain) on the 1st day of the experiment. The immunization dose was 10^2.0^ TCID_50_/ml, which was half of the normal immunization dose. The IM group was injected intramuscularly on Days 3, 5, 7, 9, and 11 after immunization, and the dose was 25 mg/kg. The IMC group was given the same dose of normal saline. No treatment was applied to either group from Days 1 to 21 of the experiment. On Day 22 of the experiment, each pig in the four groups was injected intramuscularly with 3 mL of the PRRSV JXA1 strain with a virulence of 10^5.62^ TCID_50_/ml. After challenge, the NIM group was subjected to intramuscular injection on Days 3, 5, 7, 9, and 11. The dose in the NIM group was 25 mg/kg; the NIMC group was given the same dose of normal saline. No treatment was applied to the IM group or the IMC group from Days 22 to 42.

### Detection of cytokine levels

Blood from the anterior vena cava of pigs was collected on Days 0, 14, 21, 24, 26, 28, 30, 35, and 42 of the experiment, and the samples were placed in an incubator at 37 °C for 1 h, centrifuged at 5000 rpm for 4 min, and then cryopreserved at -20 °C. Porcine IL-1β, IL-6, IL-8, TNF-α, and INF-α ELISA kits were used to detect IL-1β, IL-6, IL-8, TNF-α, and INF-α levels.

### Statistical analysis

All experiments were performed at least three times. The results are shown as the mean ± standard deviation (SD). Statistical significance was determined by Student’s t test when comparing only two groups and by one-way analysis of variance (ANOVA) when comparing more than two groups. P values of 0.05 (*), 0.01 (**) and 0.001 (***) were considered to indicate statistically significant differences.

## Results

### The maximum nontoxic concentration

**Fig. 1:**
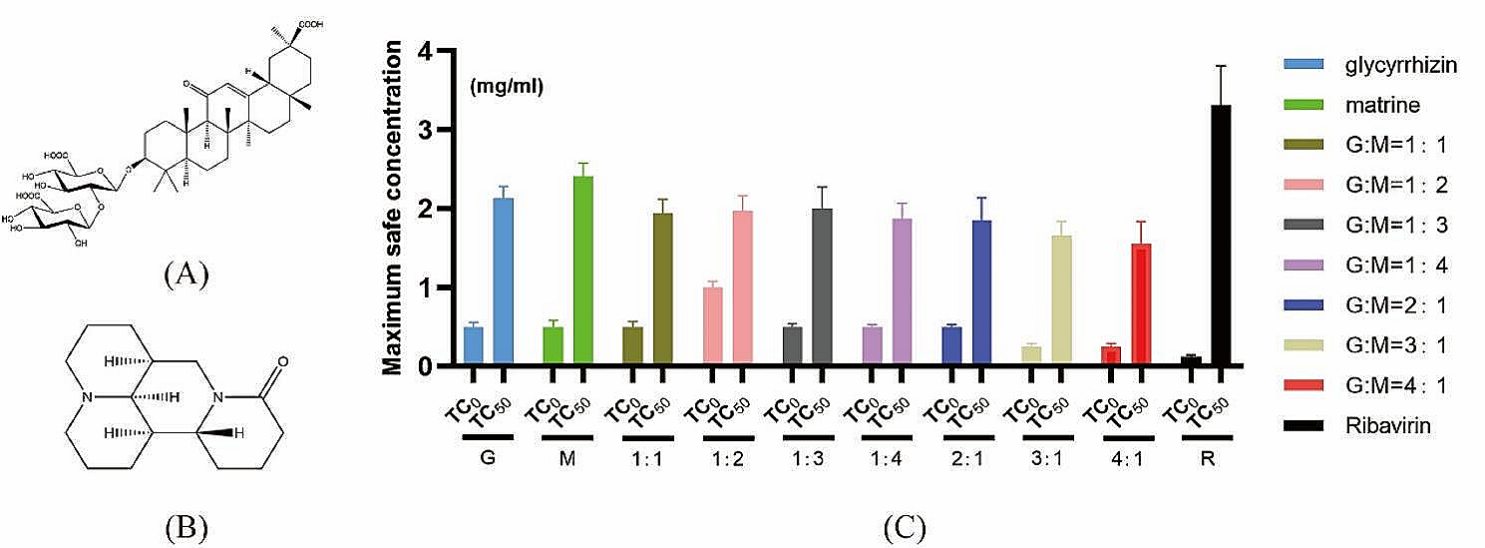
Safe concentrations of the monomeric and compound drugs in MARC-145 cells. (**A**) Chemical structure of glycyrrhizic acid; (**B**) chemical structure of matrine; (**C**) TC_0_ and TC_50_ of matrine and glycyrrhizic acid monomers and compound drugs. Note: G:M indicates the mass ratio of glycyrrhizic acid to matrine; the abbreviation for ribavirin is R. A 4:1 ratio of glycyrrhizic acid to matrine had the strongest anti-PRRSV effects

We first tested the mass ratios for the maximum safe concentration (TC_0_) and half-maximal inhibitory concentration (TC_50_) of ribavirin among the seven drugs formulated with glycyrrhizic acid (Fig. 1A) and matrine (Fig. 1B). As shown in Fig. 1C, the safe concentration of glycyrrhizic acid and matrine at 1:1, 2:1, 1:3, and 1:4 mass ratios was 0.5 mg/mL. When the mass ratio was 1:2, the safe concentration of the compound drug was 1 mg/mL; the safe concentration of the drug when the mass ratio of matrine and glycyrrhizic acid was 1:3 or 1:4 was 0.25 mg/mL; and the safe concentration of ribavirin was 0.125 mg/mL.

**Fig. 2:**
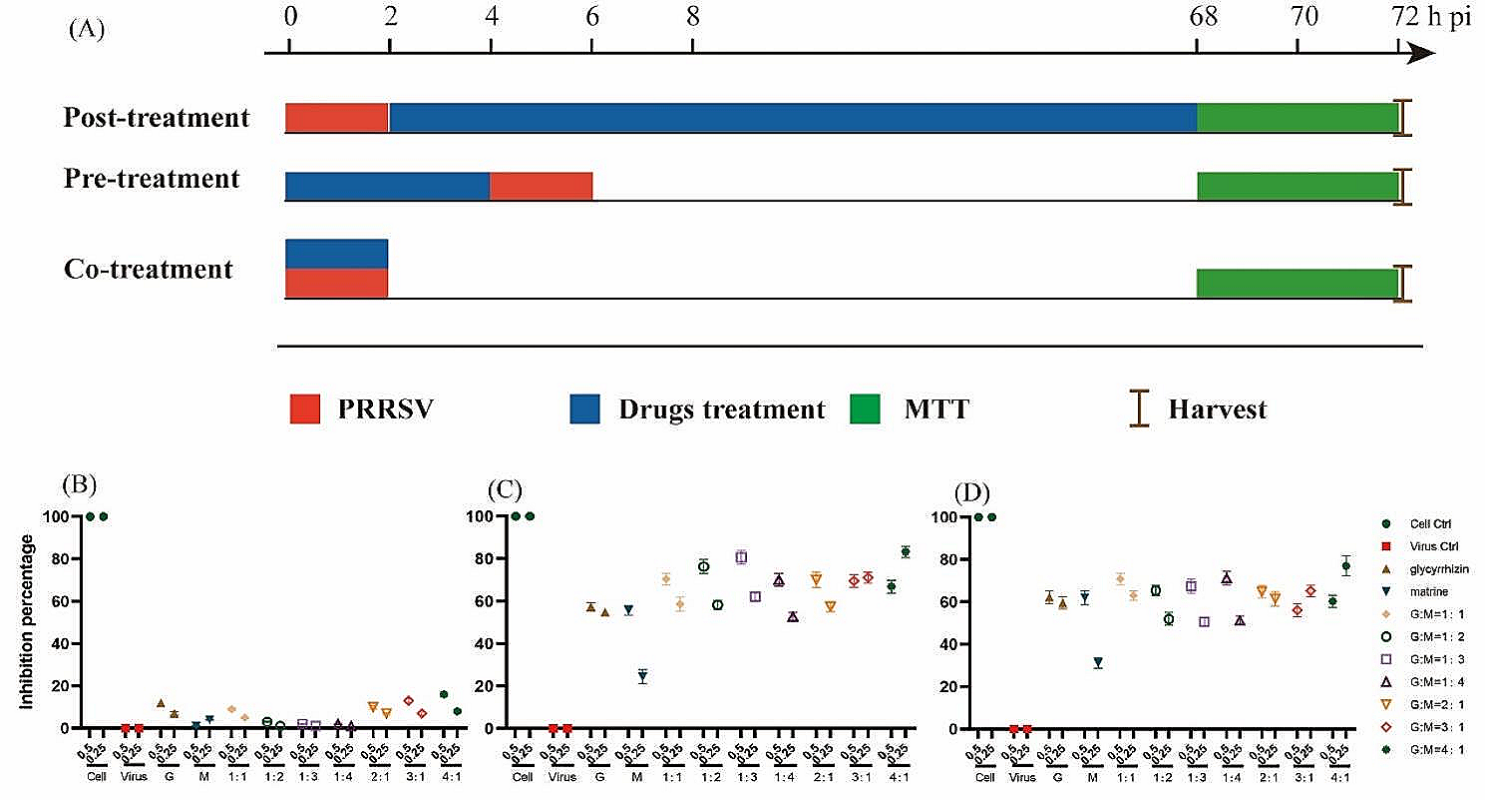
The antiviral effect of glycyrrhizic acid and matrine compounds *in vivo.* (**A**) Detailed grouping and challenge information for the time-of-addition assay; (**B**) Preventive effects of drugs on PRRSV; (**C**) Direct killing effect of drugs on PRRSV; (**D**) Therapeutic effects of drugs on PRRSV

Next, to explore the mechanism by which the drug exerts its antiviral effect, we performed a time-of-addition assay in which cells were infected with PRRSV for 2 h before (pretreatment), during (cotreatment), and after (posttreatment) PRRSV infection (Fig. 2A). The drugs were added to the cell cultures for different incubation times (Fig. 2A). The results showed that each compound had therapeutic and direct killing effects against PRRSV, and the effects of the compound drugs were better than those of the monomers but had no preventive effect (*p* > 0.05) (Fig. 2B, C and D). The glycyrrhizic acid/matrine compound had the greatest effect at 0.25 mg/mL when the mass ratio was 4:1. In the direct killing and therapeutic effect experiments, the inhibition rates of this ratio were 83.25% and 76.95%, respectively.

Ribavirin is a well-known viral RNA polymerase inhibitor that has broad-spectrum antiviral activity against infections caused by RNA viruses, including the hepatitis C virus, respiratory syncytial virus (RSV), poliovirus, and hepatitis A and B type influenza viruses (IAV and IBV). Therefore, we used 140 mM ribavirin as a positive antiviral drug control. As shown in Table 1, although the therapeutic index of ribavirin was greater than that of the other compound prescriptions, with a TI value of 31.78, its inhibition rate was lower than that of glycyrrhizic acid and matrine at a mass ratio of 4:1 and a concentration of 0.25 mg/mL. Overall, we determined that glycyrrhizic acid: matrine was the optimal compound at a ratio of 4:1 by mass and this ratio was used to continue exploring the anti-PRRSV effect of the drug.

**Table 1:** The therapeutic index of the antiviral effects of drugs

Drug	ED_50_ (mg/mL)	TC_50_(mg/mL)	Inhibition percent (%)	TI
G: M 1:1	0.166	1.946	72.47	11.723
G: M 2:1	0.190	1.853	70.13	9.752
G: M 3:1	0.173	1.656	71.11	9.572
G: M 4:1	0.122	1.549	**83.25**	**12.696**
G: M 1:2	0.233	1.966	79.28	8.436
G: M 1:3	0.244	2.005	80.63	8.217
G: M 1:4	0.248	1.875	70.15	7.560
Ribavirin	0.0985	3.13	**53.08**	**31.78**

**Fig. 3:**
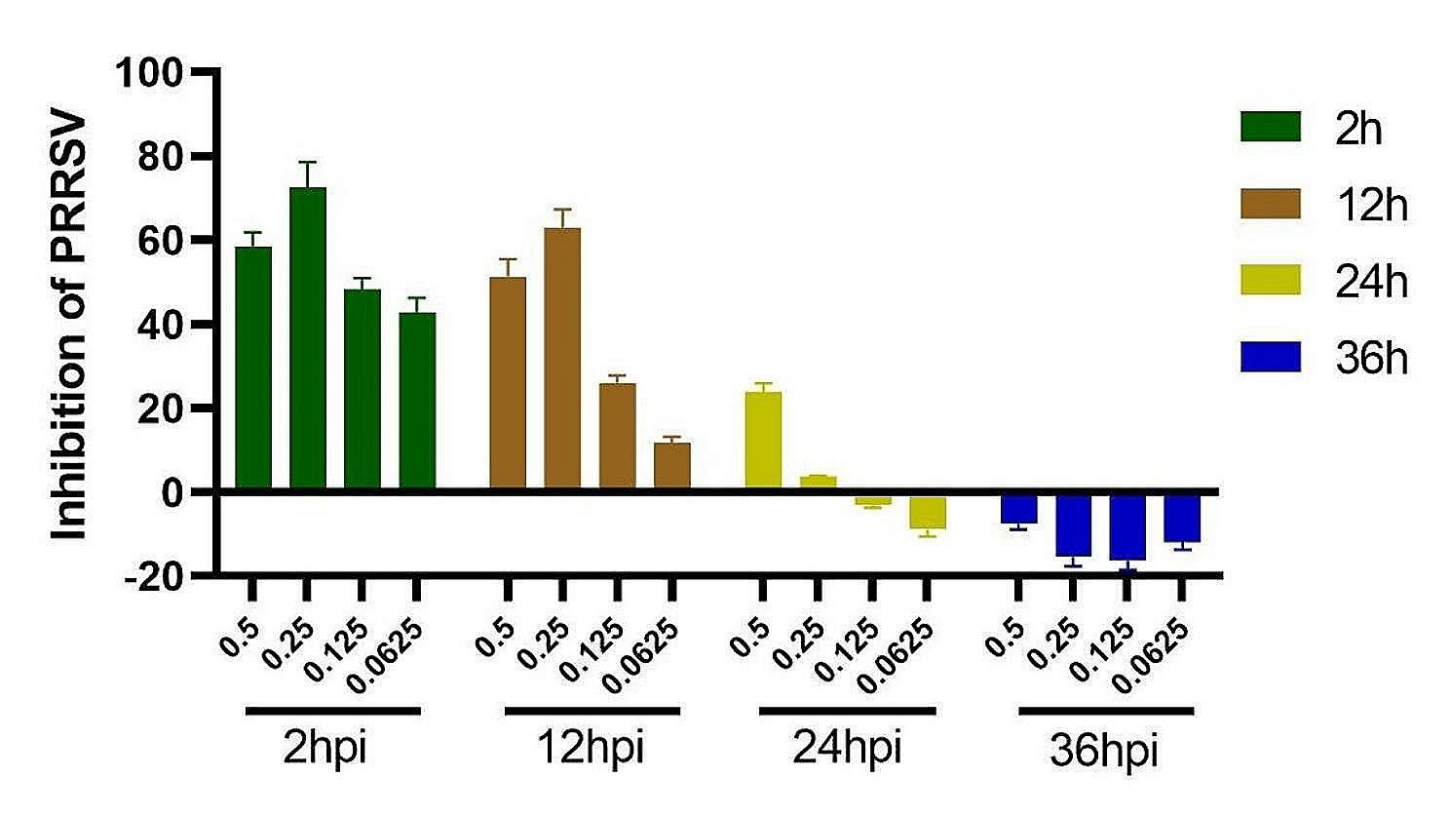
The effects of the 4:1 compound at different concentrations and different time points on proliferation

Next, we analyzed the effects of drugs at different concentrations and at different time points regarding the proliferation of PRRSV. The results are shown in Fig. 3. Between 2 and 12 h of viral infection, the drugs at all concentrations had significant inhibitory effects on the proliferation of PRRSV (*p* < 0.05). The antiviral effect of the drugs was greatest when the duration of infection was 2 h, but as the duration of viral infection increased, the effect of the drugs on the replication of the viruses decreased. When the viruses infected the cells for more than 24 h, the drugs had the greatest effects on PRRSV within the safe concentration range. There was no significant inhibitory effect on proliferation (*p* > 0.05).

MARC-145 cells were treated with glycyrrhizic acid and matrine at a mass ratio of 4:1, and the inhibition activity was determined at different concentrations and different time points. The data are from three independent experiments. The error bars denote standard errors of the means.

When the drug was used at 250 µg/mL, the TCID_50_ decreased by 1.13-fold compared with that of the virus control, and the effect of the drug on the PRRSV TCID_50_ was positively correlated with its concentration. When the drug concentration was 500 µg/mL, the TCID_50_ of the virus was 0 (Table 2).

**Table 2:** Effect of the most effective compound on the PRRSV TCID_50_

concentration	500 µg	250 µg	125 µg	62.5 µg	Virus control
TCID_50_	0	10^− 3.87^	10^− 4.36^	10^− 4.64^	10^− 5.0^

**Fig. 4:**
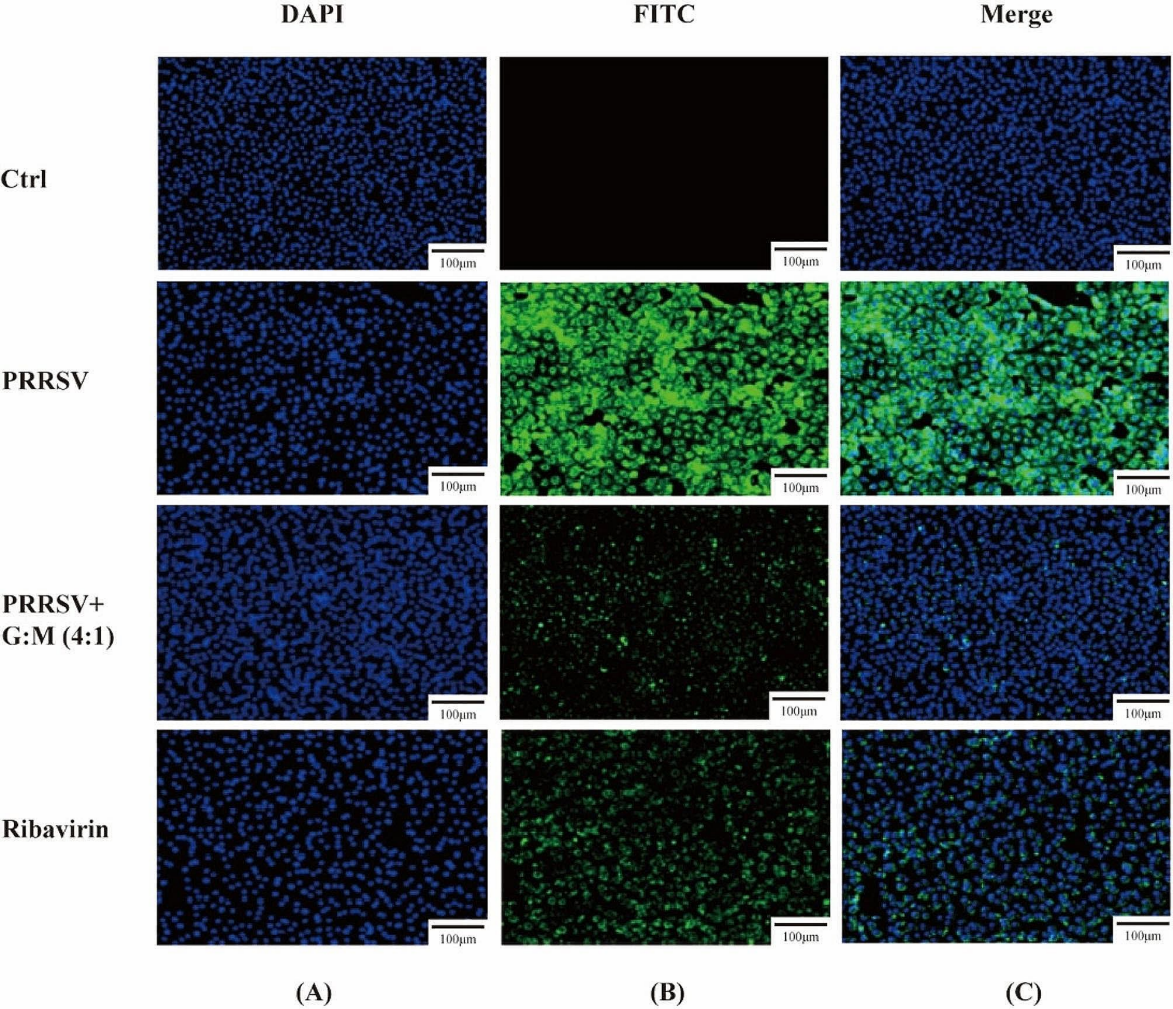
Glycyrrhizic acid and matrine (4:1) inhibited the PRRSV-N protein expression. **A**. Nuclear staining; **B**. PRRSV-N protein staining; **C**. nuclear and PRRSV-N protein staining. MARC-145 cells were treated with G: M (4:1) for 72 h. Ribavirin was used as a positive control. The cells were examined by laser confocal microscopy. Bars, 100 μm

We detected the activation of PRRSV in the cell control, virus control, 0.25 mg/mL compound group and 0.125 mg/mL ribavirin groups of MARC-145 cells at 72 h post infection (hpi). As shown in Fig. 4A, the nuclei were stained blue after DAPI application. The states of the cell nuclei in the control group and the compound drug group were similar; the nuclei were round, and the excitation colors after staining were uniform. In the virus control group and the ribavirin group, the nuclei were shrunken and had a grainy appearance. As shown in Fig. 4B, FITC-labeled N protein was stained green. The cells in the control group had no fluorescence, those in the ribavirin control group had fluorescence, those in the compound drug group had weak fluorescence, and those in the virus control group had strong fluorescence. Figure 4C shows the results of the fluorescence fusion of cells and nuclei in each group of cells. These results suggest that the compound drugs matrine and glycyrrhizinate have anti-PRRSV effects and that the effect of the compound drug is slightly better than that of ribavirin.

### The compound drug failed to exhibit a practical anti-PRRSV effect in vivo

**Fig. 5:**
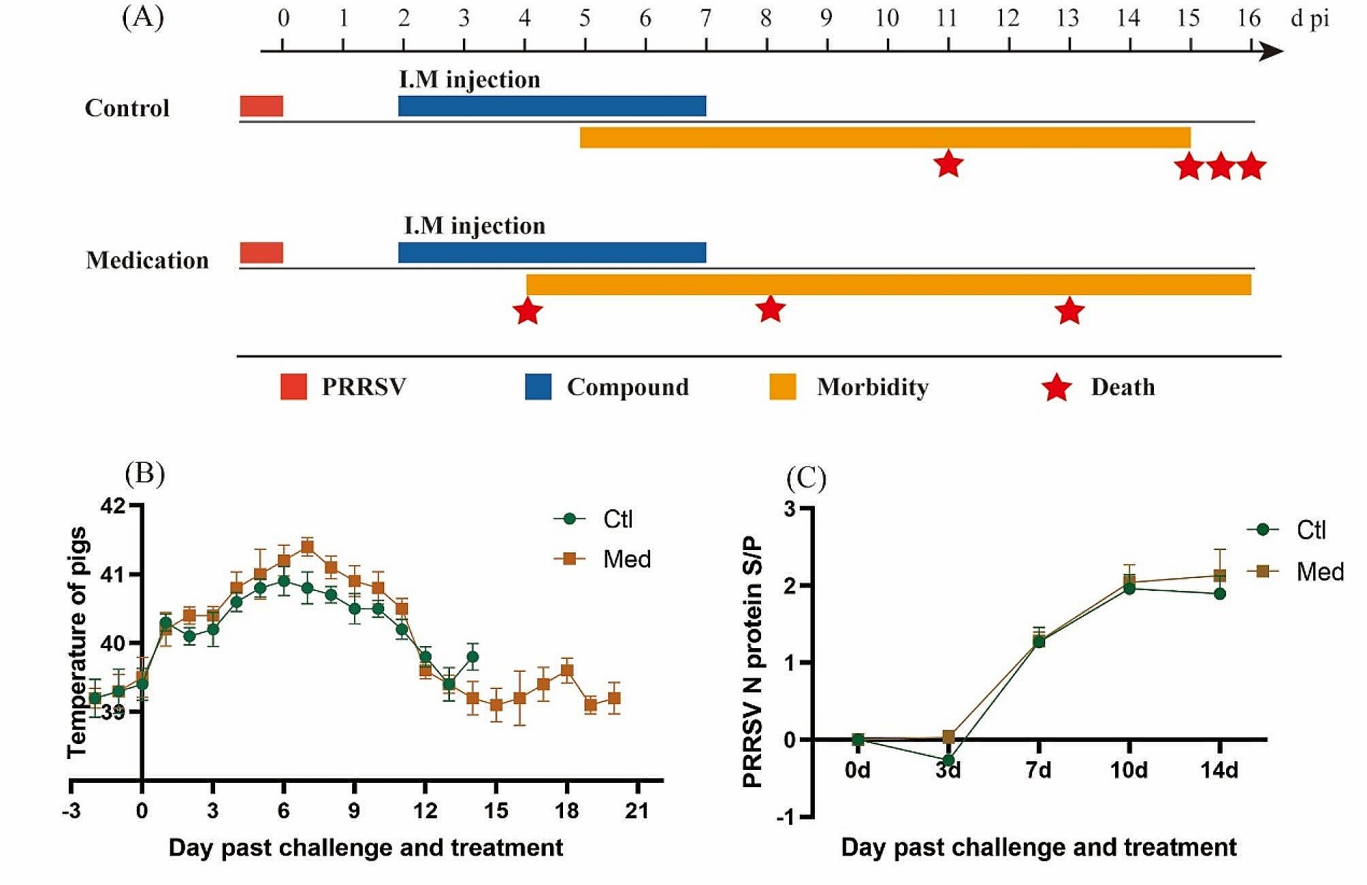
Clinical symptoms in pigs from the in vivo experiments. (**A**) Grouping, morbidity and death rates obtained from the in vivo experiment; (**B**) Temperature of the pigs; (**C**) Changes in the antibody against the PRRSV N protein in the pigs. The data are from three independent experiments. The error bars denote standard errors of the means

To examine the therapeutic effect of matrine and glycyrrhizinate compounds in vivo, we randomly divided 8 pigs into 2 groups (challenge group and treatment group). The detailed grouping and challenge information are shown in Fig. 5A. Over 21 days after the completion of infection and drug treatment, we observed and evaluated the clinical symptoms of the pigs on a daily basis; the morbidity and mortality of the pigs are shown in Fig. 5A. After challenge with PRRSV, the pigs in each group developed typical PRRS symptoms, with an incidence rate of 100%. The typical clinical symptoms of PRRS, such as decreased appetite, poor breathing, red and swollen eyelids, and depression, appeared in the control group and the medication group 3 d and 4 d after challenge, respectively.

There was no significant difference in body temperature between the control group and the medication group after challenge (*p* > 0.05). As shown in Fig. 5B, the temperature of the control group and the medication group started to rise (above 40 °C) on the 1st day of challenge, peaked on the 5th day of the challenge, and then returned to the average temperature on the 12th day of the challenge. On the 7th day of the challenge, the temperature of the challenge group was significantly higher than that of the treatment group (*p* < 0.05).

As shown in Table 3, within 21 days of the challenge, the body weight of the challenge group showed a negative increase, and the total weight gain of the medication group was 0.11 kg, which was not a significant difference (*p* > 0.05).

As shown in Fig. 5C, except for the significant difference on Day 4 (*p* < 0.05) (the antibody levels were all negative at this time), the PRRSV N antibody levels between the control group and the medication group were not significantly different (*p* > 0.05). The antibody levels were basically the same for both groups.

As shown in Fig. 5A, there were differences in mortality between the control group and the medication group. In the control group, one pig died on Day 11, and the remaining pigs died on Day 15. In contrast, in the medication group, one pig died on the Day 4, and two died on Day 8. Based on the clinical symptoms, the medication group experienced more acute death after treatment, with the mortality rate reaching 75% after the 8th day of challenge; no deaths occurred in the control group.

Overall, the results showed that the compound drug failed to exhibit a practical anti-PRRSV effect in vivo; on the contrary, it accelerated the death rate of experimental pigs infected with PRRSV.

**Table 3:** Weights of pigs within 21 d after injection of PRRSV

Group	0d	7d	14d	21d	Total weight gain	Average daily gain
Ctl	5.49	6.04	5.35	-	-0.14	-0.046
Med	5.79	5.83	6.13	5.90	0.11	0.005

Control group, Ctl; Medication group, Med;

### The compound drug does not synergistically enhance the anti-PRRSV effect of the vaccine

**Fig. 6:**
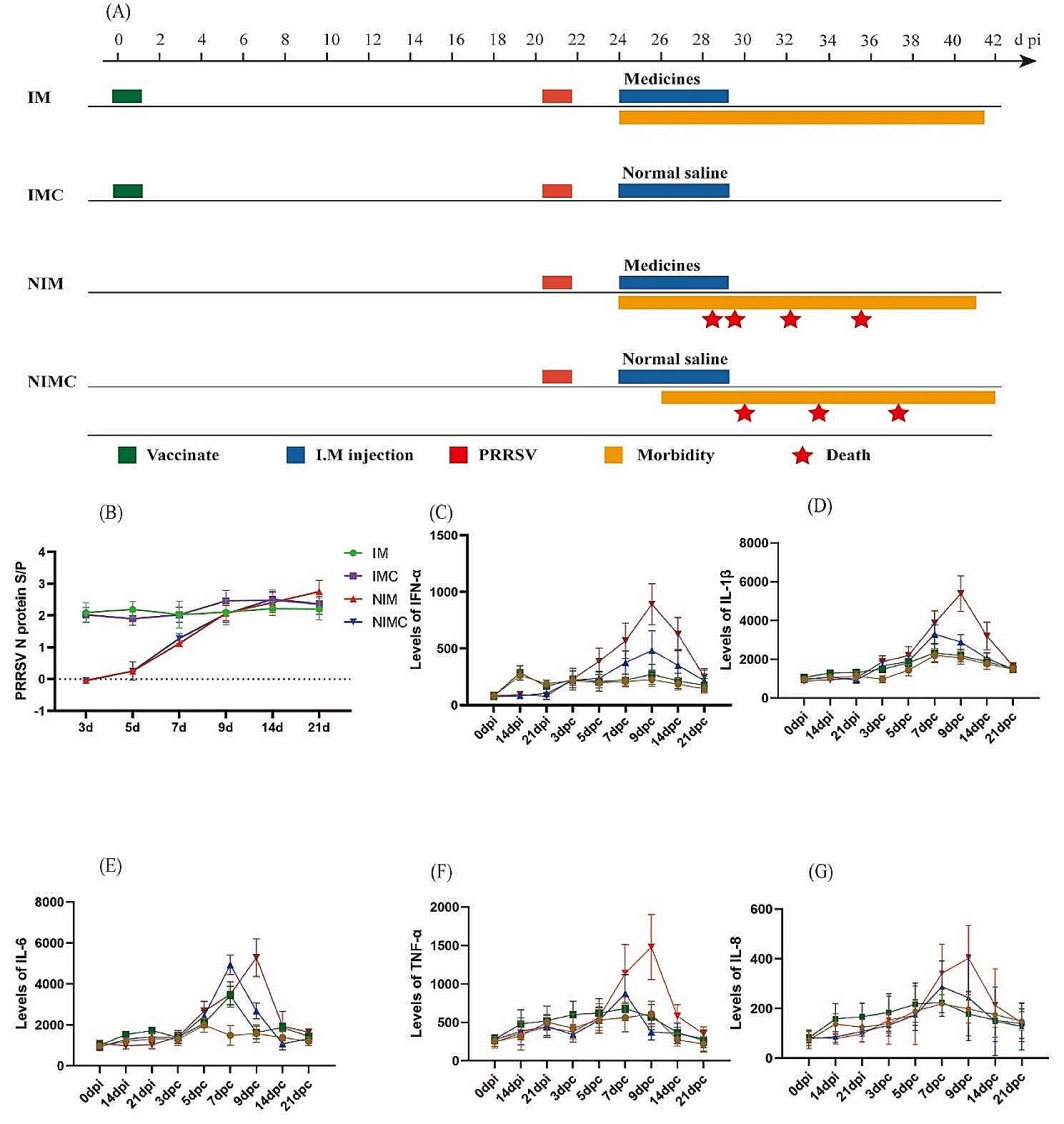
Determination of the synergistic immune effect of the compound drug vaccine. (**A**) Experimental groupings: immune drug group (IM), immunized control group (IMC), nonimmunized drug group (NIM), and nonimmunized control group (NIMC); (**B**) Antibody level of PRRSV N-protein in pigs; (**C**) Levels of IFN-α; (**D**) Levels of IL-1β; (**E**) Levels of IL-6; (**F**) Levels of TNF-α; (**G**) Levels of IL-8

There may be various reasons for the inability of the compound drug to exert an anti-PRRSV effect in vivo. We wondered whether the therapeutic effect was insufficient due to the high viral load in the body after challenge, so we used half the usual dose of the live PRRSV vaccine for immunization. In pigs, we observed whether the compound drug can exert its effect against PRRSV while providing a semiprotective effect on the animals or whether it can synergize with the vaccine virus to exert a better protective effect in vivo. We randomly divided 20 pigs into 4 groups. The detailed grouping and challenge information is shown in Fig. 6A. After PRRSV challenge, one pig in the immunized group experienced clinical symptoms on the 3rd day, whereas the other four pigs experienced no clinical symptoms; the incidence rate was 20%, and no deaths occurred. In contrast, the 5 pigs in the nonimmunized drug group (the group that was not immunized or directly challenged) all exhibited the disease symptoms on Day 3 after challenge, and the incidence rate was 100%. Death began to occur on Day 8 after challenge. The nonimmunized control group also had clinical symptoms on Day 3 after challenge, with an incidence rate of 100%. Death began to occur on the 10th day after challenge, and 3 animals died, with the mortality rate reaching 60%.

The S/P ratios of antibodies against the PRRSV N protein after 3 d of challenge are shown in Fig. 6B. The nonimmunized group did not produce antibodies after 3 d or 5 d of challenge, and the difference between the immunized and nonimmunized groups was significant (*p* < 0.05). The difference between the immunized control and control groups was not significant (*p* > 0.05). Antibodies were detected in the nonimmunized group at 7 d of challenge. After 9 d of challenge, the postinfection antibody levels of the pigs in each group were consistent, and there was no significant difference between them (*p* > 0.05).

As shown in Fig. 6C-G, there were no significant differences in the cytokine levels of the nonimmunized groups during the immunization period, whereas the levels of the inflammatory cytokines IL-6, IL-8, IFN-α, and TNF-α in the immunization group were significantly different at 14d after immunization. After challenge, the cytokine levels in the nonimmunized drug group were significantly greater than those in the nonimmunized control group at multiple time points *(p <* 0.05); the cytokine levels in the nonimmunized group were greater after challenge than those in the immunized group at all time points. The results showed that matrine and glycyrrhizic acid enhanced the inflammatory response of pigs infected with the virulent PRRSV strain and prompted the body to produce excessive inflammatory cytokines that caused body damage, preventing a therapeutic effect from being produced. Pigs did not exhibit synergistic enhancement.

## Discussion

Both matrine and glycyrrhizic acid have antiviral effects when used individual, and the antiviral effect is particularly significant when they are combined with other antiviral drugs. In this in vitro anti-PRRSV test of the compound drug, both the glycyrrhizic acid monomer and matrine monomer exhibited therapeutic and direct killing effects against PRRSV, and the anti-PRRSV effect of glycyrrhizic acid was greater than that of matrine. When the two were combined at different mass ratios, each compound drug group had a therapeutic effect and a direct killing effect on PRRSV, and the inhibition rate was greater than that of the single drug. Comparisons of the compound drugs showed that a greater glycyrrhizic acid content in the compound, produced greater inhibition of the cytopathy caused by PRRSV. The effect of matrine: glycyrrhizic acid (1:4) was greatest at 250 µg/mL and was still high. Compared with the use of glycyrrhizic acid individual, matrine and glycyrrhizic acid enhanced resistance to PRRSV in vitro. When evaluating the inhibition of PRRSV by Traditional Chinese medicine, a larger TI value, indicates safer use of the drug. In the test of the therapeutic effect of the compound on PRRSV, the maximum TI was 12.696 when the mass ratio of matrine to glycyrrhizic acid was 1:4, this was at the lowest dose. The TI of the compound drug was lower than that of ribavirin (31.78), but its anti-PRRSV inhibition rate was significantly greater than that of ribavirin, which causes minor damage to cells. In general, the rational use of drugs in combination can compensate for monomer deficiency, have a synergistic effect, and improve therapeutic efficacy.

We further studied the time- and dose-dependent anti-PRRSV efficacy of the optimal compound. The results showed that the antiviral ability was positively correlated with drug concentration within the safe concentration range. When the drug concentration was twice the safe concentration, it could completely prevent the virus from multiplying in the cells. After the drug acts on cells, it inhibits the activity of some enzymes to reduce the replication of PRRSV in the cells, thus inhibiting intracellular proliferation of the virus. In addition, the optimal compound significantly affected PRRSV replication between 2 and 12 h (*p* < 0.05), indicating a highly effective antiviral effect. After the virus had infected the cells for 24 h, however, the drugs had no significant inhibitory effect on the replication of PRRSV (*p* > 0.05). Virions proliferate in large quantities in cells over time, and the amount of drug added at this time does not reach the level required for inhibition of PRRSV replication [26]. Therefore, in actual applications, early intervention with drugs is required to achieve the desired effect.

On large-scale farms, daily weight gain, N-protein antibody levels and mortality are essential indicators for assessing the health status of pigs and determining breeding efficiency. This study used the above methods to assess the in vivo efficacy of an optimal compound for treating PRRSV. PRRSV infection can seriously affect the production performance of pigs. In this experiment, the difference in the total weight gain of the control and treatment groups was almost the same, indicating that the treatment did not improve the production performance of pigs after challenge.

Some studies have reported that PRRSV-specific antibodies could be detected in pigs approximately 7–14 days after virus infection, peaked 2–4 weeks after infection, and remained stable for several months, which is consistent with the results of our study [27]. On the 7th day of challenge, the difference in antibodies between the control and treatment groups was not significant (*p* > 0.05), indicating that the compound drug did not significantly promote the expression of specific N-protein antibodies.

In the control group, death occurred on the 11th day of challenge, and all the pigs had died by the 15th day. In the treatment group, death began to occur on the 4th day of challenge, and 2 pigs died on the 8th day; the age of death in the treatment group was thus earlier than that in the control group. Studies have reported that matrine has an inhibitory effect on immune cells such as T and B lymphocytes and peritoneal macrophages in mice [28]. Based on the clinical symptoms in the experiments, it may be that the compound drug inhibits the activity of immune cells, which accelerates the proliferation of PRRSV in pigs, causing the pigs to exhibit acute symptoms after treatment. The onset of severe symptoms can increase the mortality rate of pigs.

During the comprehensive test, based on clinical symptoms, body temperature, body weight and antibody levels, the compound drug did not achieve the expected protective effect in the pigs but instead aggravated the disease condition. This could be due to the different sources of the experimental animals, differences in health status, or the effect of the compound drug on the pigs. Upregulation of the expression of anti-inflammatory factors and downregulation of the levels of nonspecific inflammatory factors are related to the promotion of PRRSV proliferation in vivo. Therefore, we further analyzed the semiprotected pigs obtained from exposure to the compound drug to determine whether the compound drug can synergize with the vaccine in vivo.

The results showed that within the 21 days before the experiment, no pigs in the immunized group experienced symptoms, and their feed intake and temperature remained normal; after challenge, only one pig in the immunized group experienced mild clinical symptoms. The pigs in the nonimmunized group all developed the disease after challenge, and the main symptoms were depression, trembling, redness of the skin, labored breathing, and decreased feed intake as well as other symptoms in the later stage, such as unstable stances and limp limbs. Compared with the nonimmunized control group, the immunization group had great mortality, a shorter disease, earlier death after the onset of symptoms, continued emaciation after tolerance, and slower recovery of body condition. Compared with the S/P value of the PRRSV N protein antibody in the immunized group, a specific N protein antibody was detected only in the nonimmunized group on the 7th day of challenge, which increased after 9 ~ 21 d and then stabilized. The difference between the immunized group and the immunized group was not significant (*p* > 0.05).

In the early stage of PRRSV infection, PRRSV can stimulate the body to produce cytokines such as IL-1β, IL-6, IL-8, IFN-α, and TNF-α, and these cytokines protect the body by mediating the proliferation of innate immunosuppressive viruses [29]. We further analyzed the cytokine levels in the immunized and nonimmunized groups and found that the levels of inflammatory cytokines increased slightly in the immunized pigs during the immunization period under the action of the compound drug; after 14 d of immunization, the levels of IL-6, IL-8, IFN-α, and TNF-α in pigs in the immunized group were significantly greater than those in the immunized control group (*p* < 0.05). On the 9th day of challenge, the levels of all cytokines in the nonimmunized drug group were significantly greater than those in the nonimmunized control group (*p* < 0.05). The compound drugs were able to promote the production of excessive inflammatory cytokines, including in pigs infected with PRRSV. Appropriate amounts of cytokines can promote resistance to viral invasion, but excessive cytokines can damage the body and even generate cytokine storms [30]. This may also explain why the clinical reaction of the nonimmunized drug group was more severe than that of the immunized group after challenge. This phenomenon may also be related to a difference in drug’s absorption, distribution, or metabolism in the in vivo experiments or because the effect of the drug on the target was masked by other factors in the in vivo experiments.

In conclusion, although this compound drug can exert a strong treatment effect and kill PRRSV in vitro, it can cause the body to produce excessive inflammatory factors and result in body damage in pigs; as a result, this compound drug cannot be used as a treatment in pigs. The killing effect did not achieve synergistic enhancement with the live PRRSV vaccine in pigs. Therefore, in developing anti-PRRSV drugs, it is necessary to perform comprehensive drug screening, at least for solid anti-inflammatory ability, to ensure that drugs with good effects in in vitro tests also have ideal results in in vivo experiments. Our study may provide new strategies for the development of anti-PRRSV drugs.

## Conclusions

In summary, the combination of matrine and glycyrrhizic acid at different mass ratios has therapeutic and direct killing effect on PRRSV in vitro. However, it has no preventive effect, although the effect of a compound prescription with the same mass is better than that of a single prescription. In addition, in the in vitro anti-PRRSV experiment, the optimal compound mixture was matrine and glycyrrhizic acid at a mass ratio of 1:4, and the optimal dose was 0.25 mg/mL. However, the optimal compound did not show a protective effect on pigs in the PRRSV challenge experiment. The optimal compound also did not show a synergetic effect with the live PRRSV vaccine in pigs but stimulated the body to produce a more robust inflammatory response. The compound had a significant antiviral effect in an in vitro test but was not effective when used in animals. These results suggest that the existing in vitro cell drug screening models are inadequate for antiviral drug screening in vivo. New drug screening models need to be established.

## Data Availability

The data that support this study are available from the corresponding author upon reasonable request.
